# Speeding up profiling program’s runtime characteristics for workload consolidation

**DOI:** 10.1371/journal.pone.0175861

**Published:** 2017-04-27

**Authors:** Lin Wang, Depei Qian, Zhongzhi Luan, Guang Wei, Rui Wang, Hailong Yang

**Affiliations:** 1 Sino-German Joint Software Institute, Beihang University, Beijing, China; 2 School of Computer Science and Engineering, Beihang University, Beijing, China; 3 School of Data and Computer Science, Sun Yat-sen University, Guangzhou, China; Nankai University, CHINA

## Abstract

Workload consolidation is a common method to increase resource utilization of the clusters or data centers while still trying to ensure the performance of the workloads. In order to get the maximum benefit from workload consolidation, the task scheduler has to understand the runtime characteristics of the individual program and schedule the programs with less resource conflict onto the same server. We propose a set of metrics to comprehensively depict the runtime characteristics of programs. The metrics set consists of two types of metrics: resource usage and resource sensitivity. The resource sensitivity refers to the performance degradation caused by insufficient resources. The resource usage of a program is easy to get by common performance analysis tools, but the resource sensitivity can not be obtained directly. The simplest and the most intuitive way to obtain the resource sensitivity of a program is to run the program in an environment with controllable resources and record the performance achieved under all possible resource conditions. However, such a process is very much time consuming when multiple resources are involved and each resource is controlled in fine granularity. In order to obtain the resource sensitivity of a program quickly, we propose a method to speed up the resource sensitivity profiling process. Our method is realized based on two level profiling acceleration strategies. First, taking advantage of the resource usage information, we set up the maximum resource usage of the program as the upper bound of the controlled resource. In this way, the range of controlling resource levels can be narrowed, and the number of experiments can be significantly reduced. Secondly, using a prediction model achieved by interpolation, we can reduce the time spent on profiling even further because the resource sensitivity in most of the resource conditions is obtained by interpolation instead of real program execution. These two profiling acceleration strategies have been implemented and applied in profiling program runtime characteristics. Our experiment results show that the proposed two-level profiling acceleration strategy not only shortens the process of profiling, but also guarantees the accuracy of the resource sensitivity. With the fast profiling method, the average absolute error of the resource sensitivity can be controlled within 0.05.

## Introduction

Workload consolidation refers to running multiple programs simultaneously on one server [1]. As a tradeoff between workload performance and resource utilization, workload consolidation is very popular in the clusters and data centers. Though improving the resource utilization [2], workload consolidation introduces new challenges to the performance of individual program and the throughput of the whole system. First, co-running programs tend to compete for the shared resources, resulting in performance degradation of some programs [3–6]. Second, the co-running programs may interfere with each other because of the different runtime behaviors, which makes the performance prediction difficult [7–11]. Third, a co-running program may be blocked due to the shortage of a single shared resource, which ruins the throughput of the entire server or cluster [12].

In order to get the maximum benefit from workload consolidation, the programs allocated to the same server should have no or less resource demand conflict. To achieve this goal, the scheduler for workload consolidation needs to clearly understand the program runtime behavior.

For the purpose of sufficient understanding of the program runtime behavior, we propose a novel metrics system to comprehensively depict the runtime characteristics of programs. The metrics system includes (1) the resource usage of the program, and (2) the resource sensitivity of the program. Resource usage refers to the amount of the resource required by the program execution, and resource sensitivity is defined as the program performance degradation when some of the required resources are not available. This metrics system can help in predicting the performance degradation of a program when it is co-running with other programs, in safe and efficient scheduling of co-running programs, and in improving program performance, system throughput and resource utilization at the same time.

Though the resource usage of a program can be obtained by common performance analysis tools, acquisition of the resource sensitivity is not so straightforward. To our best knowledge, there is no specific tools to measure the resource sensitivity, and the resource sensitivity obtained by existing methods is not able to reflect the sensitivity to multiple resources. The simplest and the most intuitive way to obtain program’s resource sensitivity is to run the program in an environment with controllable resource provision and record the performance of program under different levels of resource conditions. To achieve this, we take advantage of Cgroups [13] to set up a control group with tunable resources. Cgroups is a mechanism within Linux kernel which provides the ability of allocating resources among user-defined groups. The program being profiled is put into the control group for execution under various resource conditions. The program performance obtained from execution in the control group is called the performance with resource restriction. According to its definition, the resource sensitivity of a program is calculated as the ratio of the performance with resource restriction to the performance without resource restriction. Sensitivity acquisition is time-consuming, especially when multiple resources are involved and the resources are controlled in fine granularity. In that case, a great number of program executions have to be conducted to get the program resource sensitivity under all possible resource conditions.

In order to speed up the process of obtaining the resource sensitivity of a program, we propose a fast profiling method in this paper to reduce the number of program executions required for profiling the resource sensitivity. Our method consists of two levels of profiling acceleration strategies. First, taking advantage of the maximum resource usage information, we set up the maximum resource used as the upper bound of the resource level, called resource ceiling, in the profiling process. By doing so, we only need to conduct the experiment up to the resource ceiling, eliminating profiling executions over the whole range of the available resources. This will reduce the number of profiling executions significantly. Secondly, by means of a prediction model supported by interpolation we can cut the number of the experiments even further. Resource sensitivity at sparse points over the resource range are obtained by real profiling executions, while the resource sensitivity values at other denser points can be calculated by the prediction model. These two profiling acceleration strategies have been implemented and applied to our practice in profiling program runtime characteristics. The experiment results show that the proposed fast profiling method can reduce the number of experiments while still guaranteeing required accuracy of resource sensitivity.

## Motivation and challenges

### Necessity of understanding the program run-time characteristics

In order to fully exploit the advantages of workload consolidation [14, 15] while still maintaining a satisfactory program performance, programs with less resource demand conflict should be co-scheduled to the same server. For the purpose of optimal scheduling in the case of workload consolidation, the task scheduler has to fully understand the program’s runtime characteristics [16].

**Fig 1:**
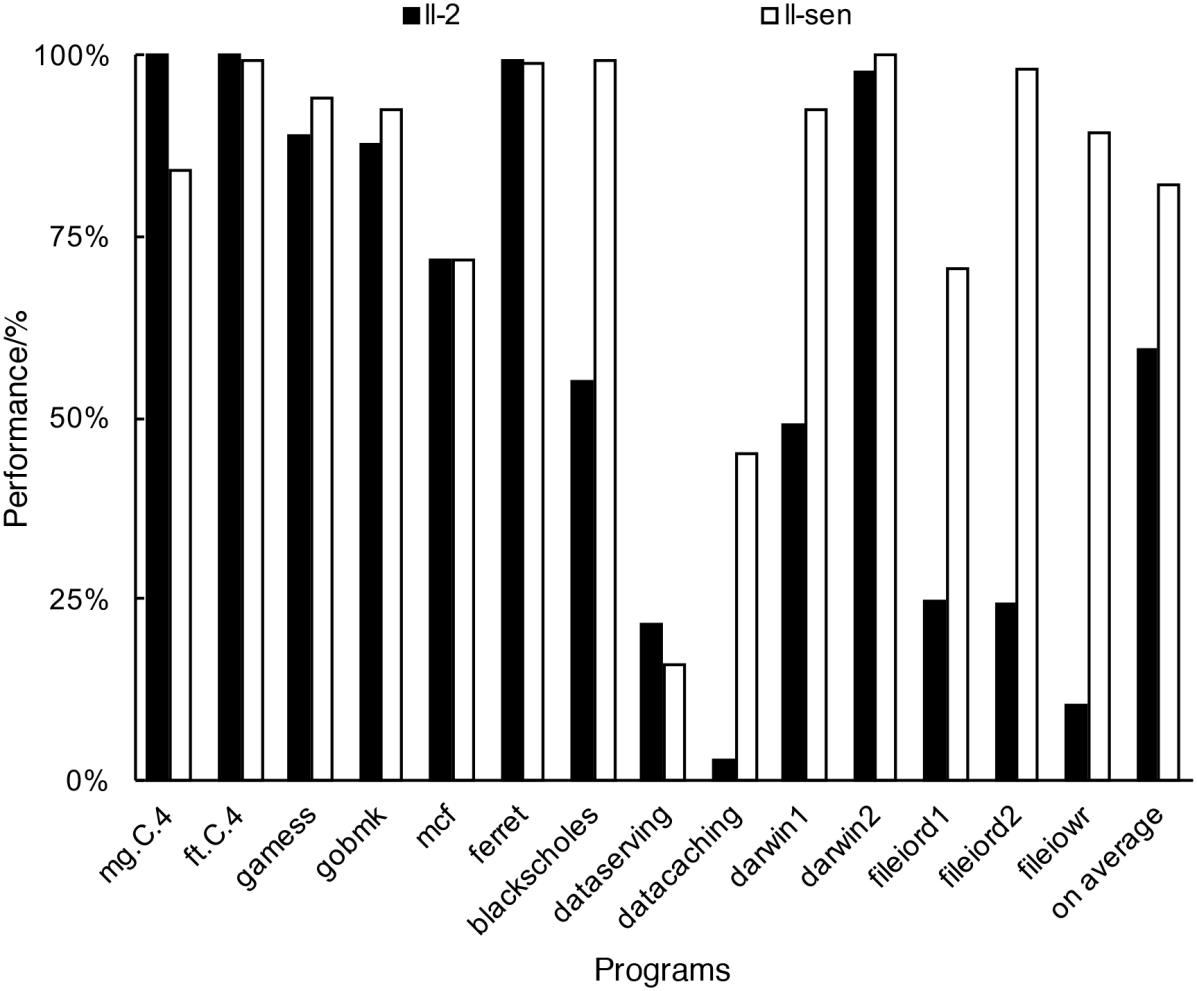
Example of metrics system advised scheduling. Fourteen programs are scheduled to four servers using two scheduling strategies. Compared with ll-2, by making use of ll-sen the average performance of programs improves 22.7%.

We define a multi-resource multi-perspective metrics system to depict the runtime characteristics of a program. Five different resources are selected as shared resources which are competed by co-running programs. Those resources include CPU, disk read bandwidth, disk write bandwidth, memory capacity and network bandwidth. Two perspectives, resource usage and resource sensitivity, are used to describe the program runtime behavior on the resource usage. After labeling the programs with this multi-resource multi-perspective metrics, the task scheduler can do a better job in metrics system advised scheduling than the traditional least load scheduling. In order to understand the importance of using the resource usage and resource sensitivity in scheduling, we realized an example of metrics system advised scheduling shown in Fig 1. Fourteen programs selected from NAS Parallel Benchmark [17], SPEC CPU2006 [18], PARSEC [19], Cloudsuite [20] and SysBench [21] are scheduled to four servers using two scheduling strategies. ll-2 is the most common least load scheduling strategy [22] which maps the programs to the server according to their CPU and memory usage. ll-sen is the scheduling strategy based on the multi-resource and multi-perspective metrics system, which maps the programs by considering the resource usage and resource sensitivity over the five shared resources mentioned above. We define the performance of running-alone program as 1, which is the case of using the resource exclusively. We find that different scheduling strategies result in quite different performance. When using ll-sen in scheduling, the performance of most of the programs such as games, gobmk, blackScholes, Data Caching, darwin1, fileiord1, fileiord2 and fileiowr are improved compared with the case of using ll-2, only mg.c.4, ft.c.4 and ferret suffer a slight decrease of performance. Note those programs with performance loss are all cpu-demanding programs, it is because when doing the mapping decision, ll-sen considers both the usage and sensitivity of five kinds of resources. By sacrificing a little performance of CPU-intensive programs, ll-sen can significantly increase the performance of other kinds of programs. This example shows that scheduling considering both the resource usage and the resource sensitivity can improve the performance of workload consolidation.

### Difficulty in profiling the resource sensitivity

We define the resource sensitivity of a program at a specific resource condition as the ratio of the performance obtained with the restricted resources to the performance without resource restriction, which is formulated by Eq (1).

$$\begin{array}{c} S_{C[i],Dr[j],Dw[r],M[s],N[t]}=\frac{P_{C[i],Dr[j],Dw[r],M[s],N[t]}}{P_{R[res-unlimited]}} \end{array}$$ (1)

**Fig 2:**
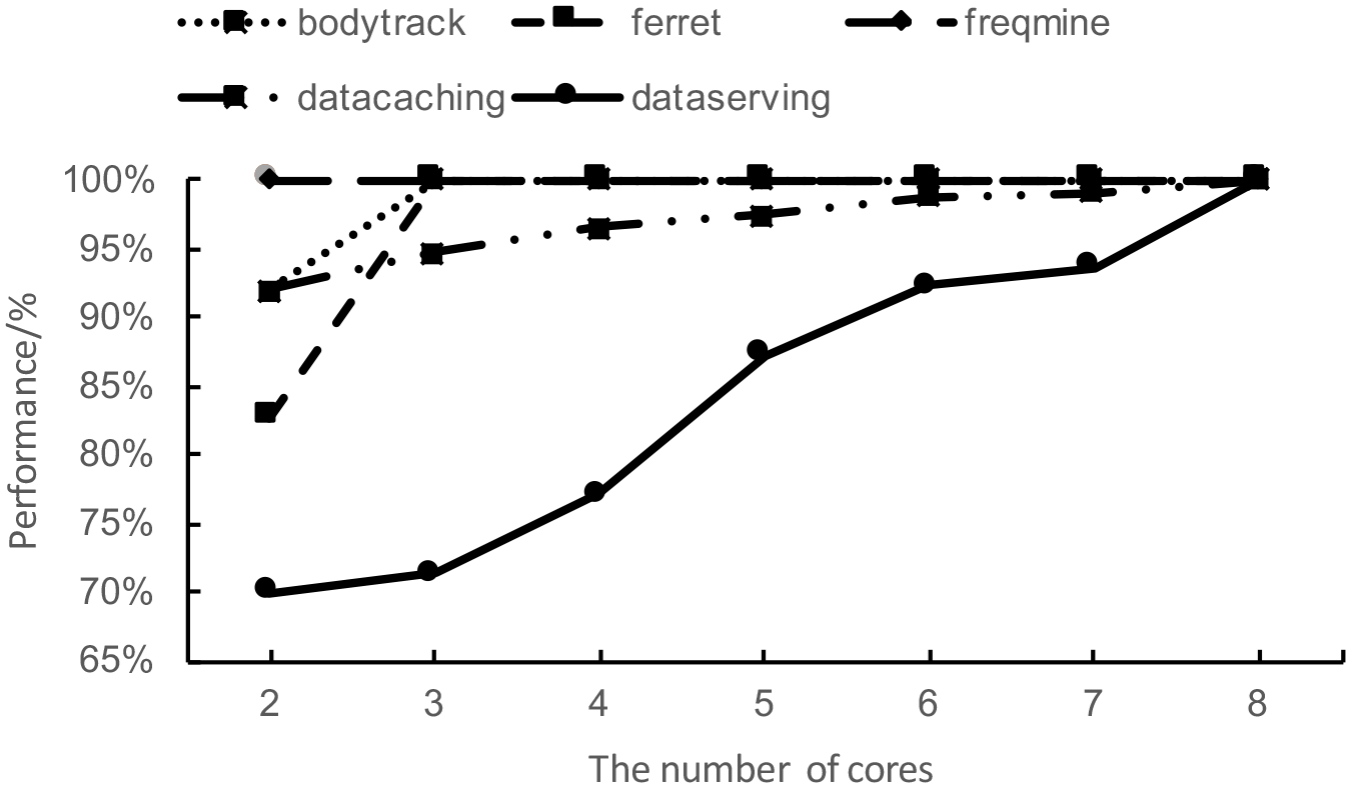
Program sensitivity curve on CPU. With one kind of controllable resource, the resource sensitivity of the program is a curve.

**Fig 3:**
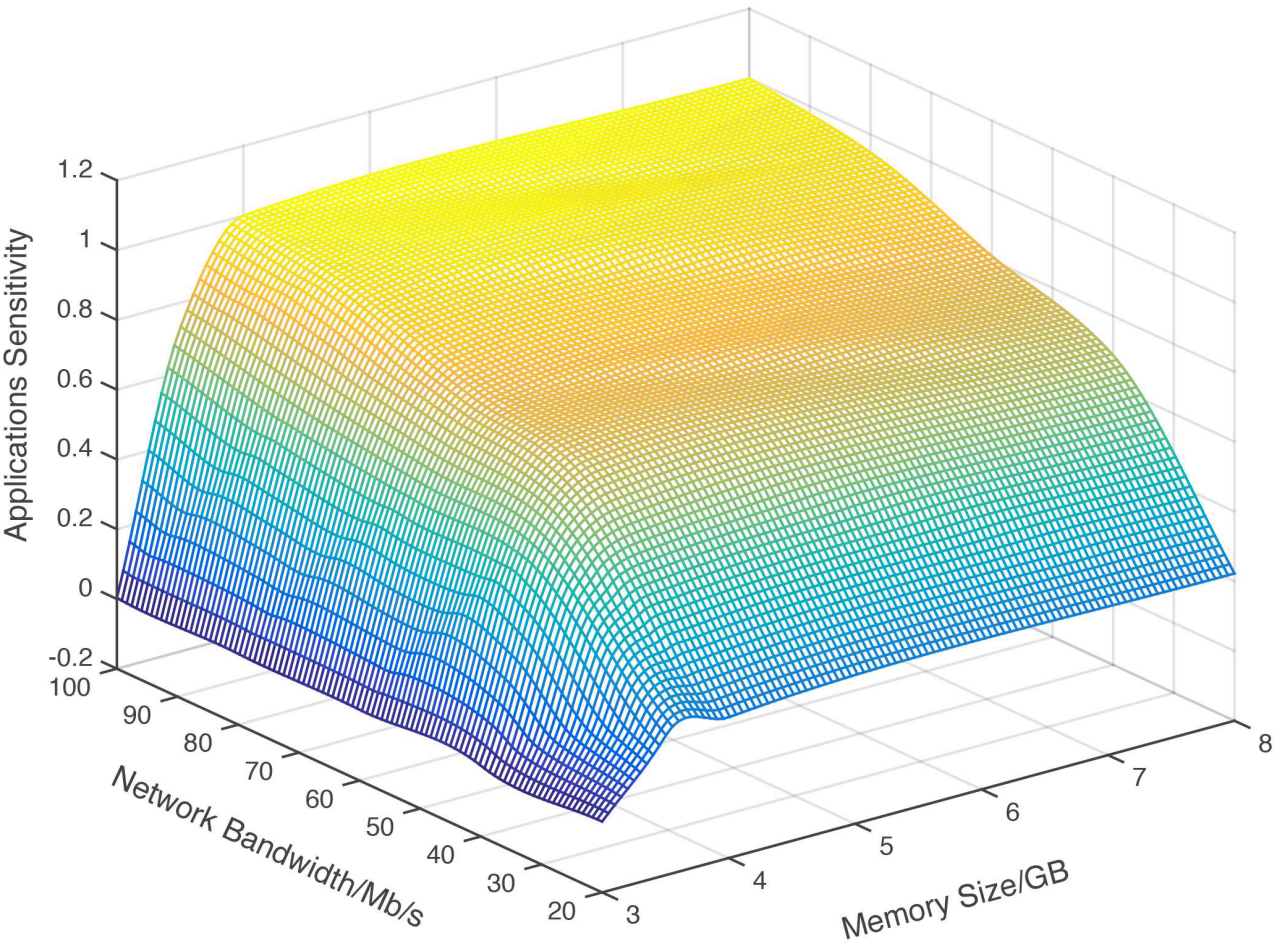
Data caching’s sensitivity on memory & network bandwidth. With two kind of controllable resource, the resource sensitivity of the program is a surface.

Here, *P*_*R*[*res*−*unlimited*]_ is the program performance without resource restriction, that is, the performance achieved with the maximum resource. *P*_*C*[*i*],*Dr*[*j*],*Dw*[*r*],*M*[*s*],*N*[*t*]_ is the program performance under a specific resource condition of the number of CPU cores (C[i]), the specific disk read bandwidth (Dr[j]), the specific disk write bandwidth (Dw[r]), the size of memory (M[s]) and the specific network bandwidth (N[t]). It is easy to perceive that with one kind of controllable resource, the resource sensitivity of a program is a curve. Fig 2 shows an example of resource sensitivity curve against CPU. Five programs selected from the benchmarks PARSEC and CloudSuite are profiled and the results are shown in Fig 2. It is also easy to understand that the resource sensitivity against two resources forms a surface, Fig 3 shows the sensitivity surface of the program Data Caching from CloudSuite against varied memory capacity and network bandwidth. In a more general case, the program resource sensitivity against more than two varied resources forms a hyperplane. In the case of above five-resource metrics system, the resource sensitivity is a 5-dimensional hyperplane.

We have developed our method for profiling the resource sensitivity of a program against the five shared resources. This method does not need the knowledge of source code of the profiled program, and does not rely on the programming environment. It is based on Linux kernel with Cgroups. We set up a control group by Cgroups and control the amount of resources allocated to the control group for program execution. Each resource is divided into multiple levels, from the minimum to the maximum of the resource capacity. The program execution environment is adjusted by providing all possible combinations of resources at different resource levels.

**Fig 4:**
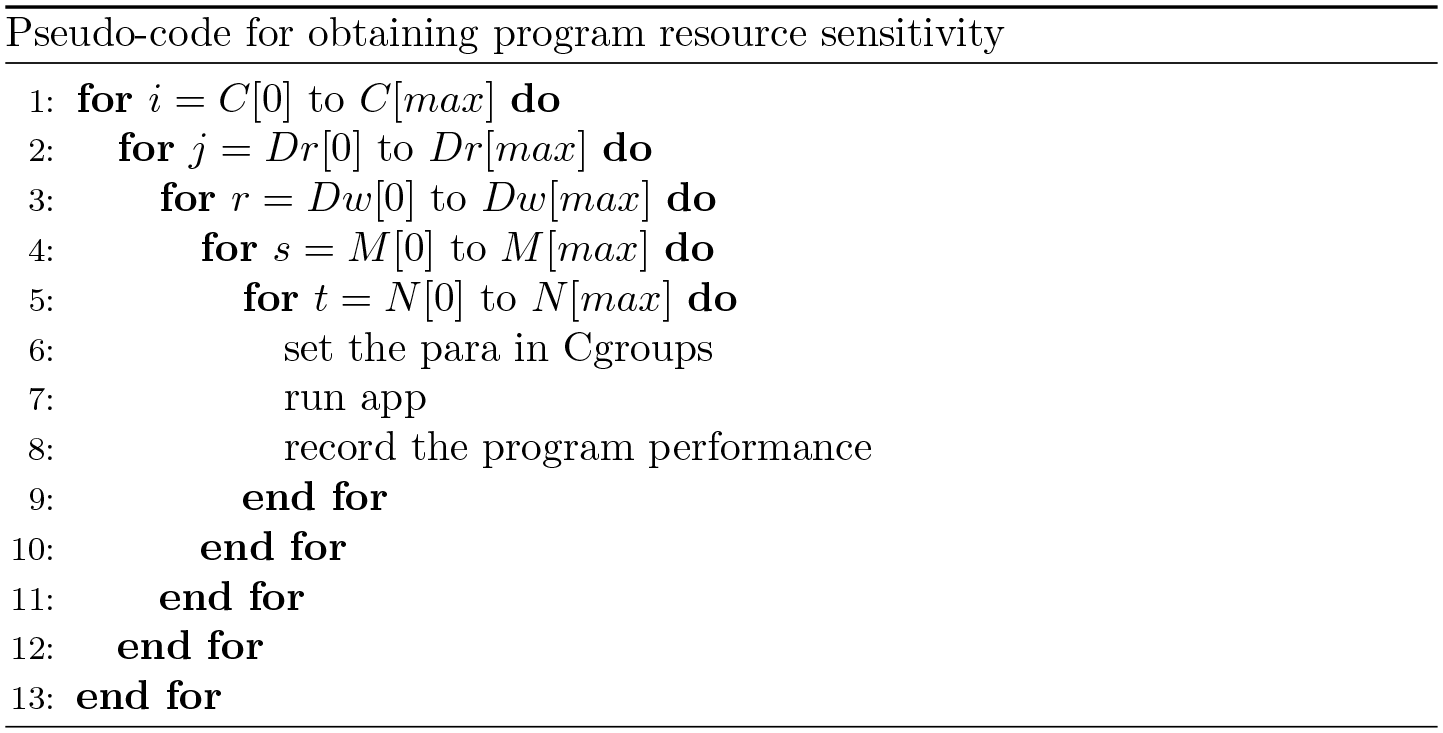
Pseudo-code of obtaining program resource sensitivity. An array is used to describe all resource restriction on one resource, and five arrays represent five resources respectively. The experiment will execute with nesting iterations over each of the resource, and record the performance of the program.

The simplest and the most intuitive way to obtain the sensitivity hyperplane of a program is to let the program run in the control group under the all possible resource conditions and records the performance achieved in each resource condition. The pseudo-code to perform the profiling executions is in Fig 4. An array is used to describe all resource restriction on one resource, and five arrays represent five resources respectively. The experiment will execute with nesting iterations over each of the resource, and record the performance of the program.

However, if the resource is adjusted in fine granularity, the iterations will go through many steps. For example, if the five resources are controlled in 7, 24, 21, 13 and 17 levels respectively, taking the brute force approach, the number of iteration steps to complete profiling is 7*24*21*13*17. We take the program Data Serving as an example and use the throughput as the performance criteria. It takes 550 seconds to accomplish one execution of Data Serving with non-restricted resources. With restricted resources, it will take more than 550 seconds to complete one step of the profiling process. Therefore, with the brute force method, the time required to complete the profiling process will be longer than 550*7*24*21*13*17 = 428828400 seconds (i.e., 119119 hours!). It can be seen that getting the resource sensitivity hyperplane of a program is a very tedious work, especially when multiple resources are involved and each resource is controlled in fine granularity. We need to find more efficient approaches to obtain the resource sensitivity hyperplane while still keeping reasonable accuracy of the profiling result.

## The fast profiling method

From the above discussion we can learn that the time required for profiling the resource sensitivity increases with the increase of the shared resource types and the resource control levels. The cost of going through all resource level combinations by means of the brute force method will be unacceptable. An intuitive way of speeding up the profiling process is to reduce the number of iteration steps in the profiling execution. In this paper, we propose a fast profiling method which implements two levels of optimization: (1) narrowing the scope of experiments and eliminating the program execution in the resource conditions above the maximum resource usage, and (2) executing the program at only sparse points and calculating the sensitivity values of other points by interpolation.

### Profiling acceleration based on resource ceiling

The resource ceiling of a program is defined as the resource usage of the program execution. In our study, it is denoted as

$$\begin{array}{c} resource\;ceiling=(R_{CPU-ceiling},R_{Dr-ceiling},R_{Dw-ceiling},R_{M-ceiling},R_{N-ceiling}) \end{array}$$ (2)

In many cases, the resource ceiling is lower than the physically available resources. Adding more resources beyond the resource ceiling is useless and will not improve the performance of the program execution. This fact can be utilized to eliminate unnecessary profiling execution. We propose a fast profiling strategy using the resource ceiling instead of the maximum resource capacity as the upper bound of the profiling scope. Profiling execution in resource conditions beyond the resource ceiling can be avoided, thus the process of determining the resource sensitivity can be accelerated. Taking the program Data Caching as an example, the actual maximum memory usage of Data Caching is 4 GB, so we take 4GB instead of 8GB as the memory resource ceiling of this program. The resource ceiling based profiling algorithm includes four steps.

**Fig 5:**
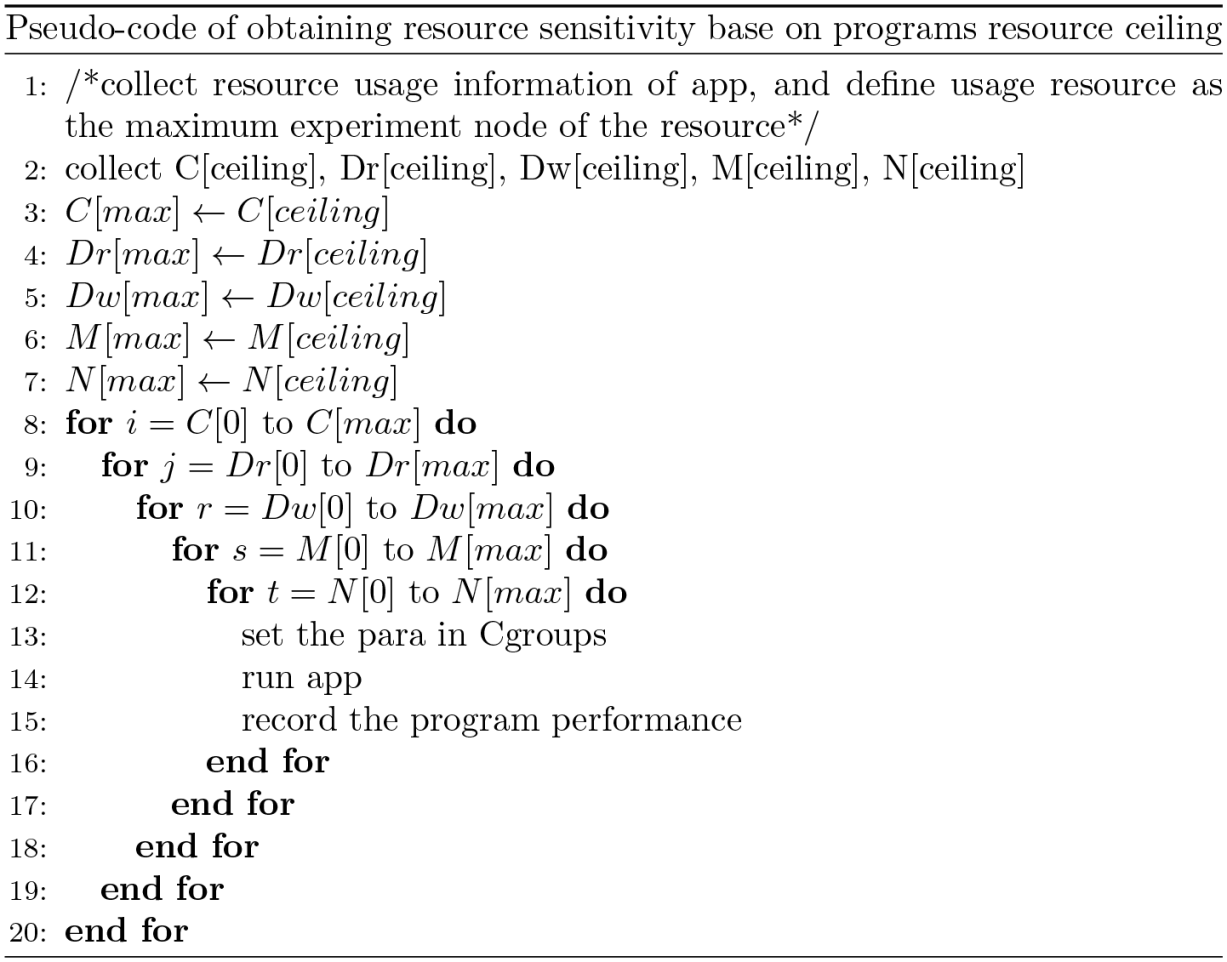
Pseudo-code of obtaining the resource sensitivity base on program’s resource ceiling. Using the resource ceiling of a program to narrow the scope of the restricted resources. Compared with the simplest method, profiling acceleration based on resource ceiling takes the resource usage as the max point.

1. The resource ceiling is obtained by running the program with unrestricted resources, (i.e., the maximum resources available on the server). This is realized by running the program alone on a server, recording its performance, and monitoring its resource usage using the performance monitoring tool collectl [23]. The maximum resource usage monitored will be used as the resource ceiling in the following steps. The performance achieved in this execution is recorded at the same time and used as *P*_*R*[*res*−*unlimited*]_ in Eq (1).
2. Set up the experiment points. For each resource, the resource ceiling value obtained in step 1 is used as the upper bound of the controlled resource. The lower bound of the resource is set to be 20% of its capacity. Then we uniformly set the resource level points between the lower bound and the upper bound with a predefined resource increment. The resource increments are denoted by *D*_*CPU*_, *D*_*Dr*_, *D*_*Dw*_, *D*_*M*_, and *D*_*N*_, representing the resource level change of CPU, disk Read, disk write, memory and network, respectively in each experiment step. Taking the CPU resource as an example, the experiment points between the lower and upper bound are set as (*R*_*CPU*−*lowerbound*_, *R*_*CPU*−*lowerbound*_ + *D*_*CPU*_, *R*_*CPU*−*lowerbound*_ + 2 *D*_*CPU*_, ⋯, *R*_*CPU*−*upperbound*_). An experiment execution is required at each point. The process of obtaining the resource sensitivity is organized as nesting loops shown in Fig 5. The number of nesting loops equals to the number of different resources, and the number of iterative executions within each loop equals to the number of resource levels between the lower bound and the upper bound of the corresponding resource.
3. Perform the profiling process with iterative executions of the program over each resource. The pseudo-code of the algorithm for profiling is shown in Fig 5. It executes the program at every resource level point in the loop and records the corresponding program performance at each point as *P*_*C*[*i*],*Dr*[*j*],*Dw*[*r*],*M*[*s*],*N*[*t*]_ in Eq (1). This process continues until the iterations over all resources are finished.
4. Calculate the program resource sensitivity using Eq (1).

It can be learned from the above algorithm that the time spent on conducting the profiling executions is proportional to the number of resources and the size of resource level increment. With a fixed size of the resource increment, reducing the range of resource will reduce the number of profiling executions. Therefore, using the resource ceiling to limit the scope of resource will speed up the process of resource sensitivity profiling.

### Interpolation-based profiling acceleration

In order to get more precise resource sensitivity, profiling at denser resource level points is preferred. On the other hand, selecting dense resource level points means more steps in the iterative execution and therefore prolongs the profiling process. One intuitive thought in further reducing the profiling time is to use the sensitivity prediction based on interpolation technique. The concept is to select a small number of points as the sample set and execute the program at those points. The results obtained from the executions on the sample set are used to generate the fitting functions by interpolation. The fitting functions are then used as a prediction model to calculate the resource sensitivity values at the points where no real profiling execution has been conducted. So the sensitivity prediction can be defined as taking a small number of points with known sensitivity value as the input to predict other points with unknown sensitivity value. In order to effectively implement the interpolation-based acceleration, we have to answer two questions: how to quickly generate the sampling set? and how to generate a fitting function which fits the real sensitivity well?

#### A quick method for generating the sampling set

**Fig 6:**
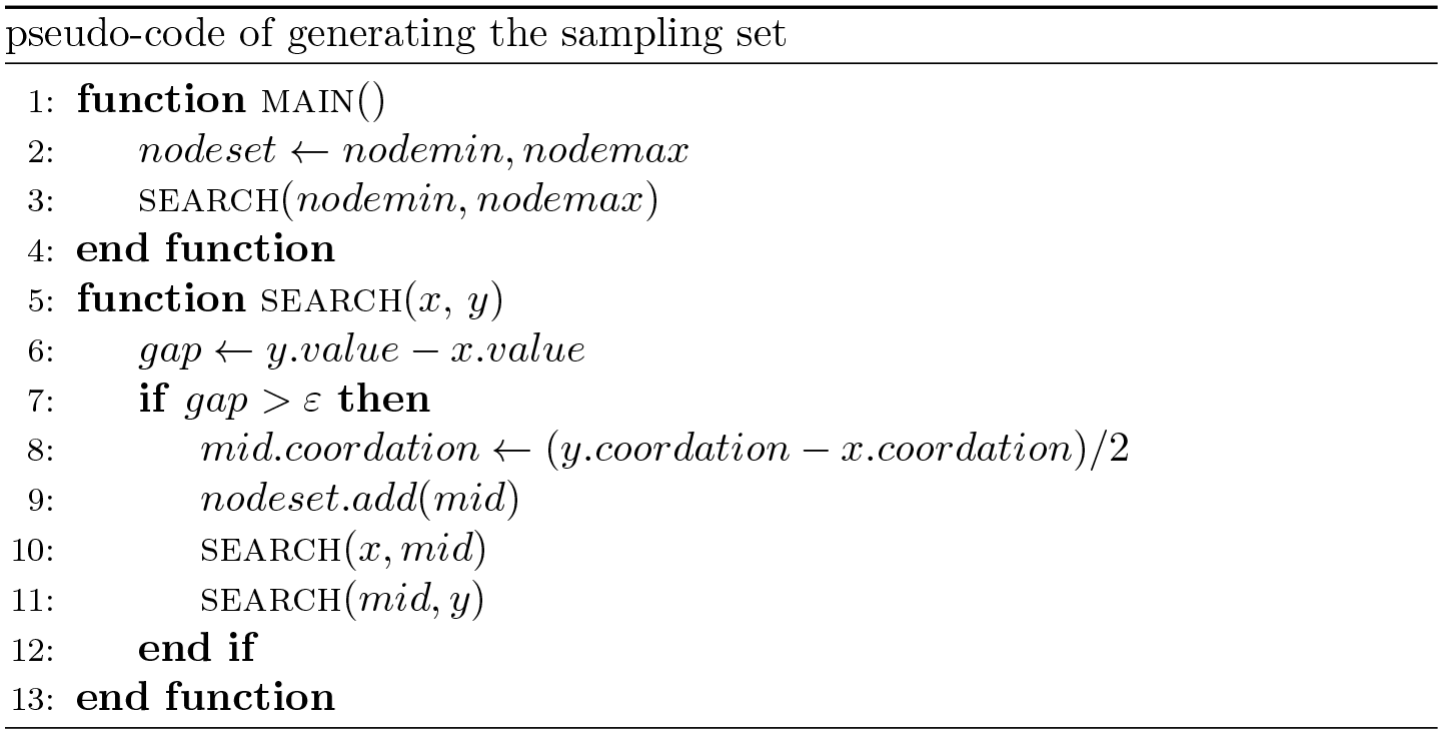
The pseudo-code of generating the sampling set. Putting the minimum point and the maximum point (resource ceiling) into the sampling set and execute the program with resource restriction at these two points. If the difference in resource sensitivity at these two points is smaller than a predefined value *ε*, the job is done, otherwise, find the mid-point between the minimum point and the maximum point and form two subsets {minimum point, mid-point} and {mid-point, maximum point}.

A method similar to binary search [24] is adopted for generating the sampling set of the five resources. We name it binary recursive search. Initially the sampling set is empty. We put the minimum point and the maximum point (resource ceiling) into the sampling set and execute the program to get the resource sensitivity at these two points. If the difference between the resource sensitivity values at these two points is smaller than a predefined value *ε*, the job is done. Otherwise, find the mid-point between the minimum point and the maximum point, put the mid-point into the sampling set and form two search subsets minimum point, mid-point and mid-point, maximum point. Then perform the binary search recursively on those two subsets. We can adjust the value of *ε* to control the time of generating the sampling set. Usually, a larger *ε* will result in a short time. The pseudo-code of the method is shown in Fig 6.

#### Generating the interpolation functions and calculating the unknown values

The basic idea of interpolation [25] is the following: assume function *y* = *f*(*x*) is defined on [*a*, *b*], and the function’s value on *a* ≤ *x*_0_ < *x*_1_ < ⋯ < *x*_*n*_ ≤ *b* are *y*_0_, *y*_1_, ⋯, *y*_*n*_, then build a simple function *P*(*x*), making *P*(*x*_*i*_) = *y*_*i*_(*i* = 0, 1, ⋯, *n*) established, then *P*(*x*) is the interpolation function of *f*(*x*). Making use of *P*(*x*), the value at a new point *x* can be calculated, and the value *P*(*x*) is an approximated value of the function *f*(*x*) at the point *x*.

We choose piecewise cubic Hermite interpolation as the interpolation function. Compared with other interpolation functions such as Spline interpolation, the Hermite interpolation matches better to the resource sensitivity curve, which is smooth and monotonicity. The basic definition of 1-dimensional piecewise cubic Hermite interpolation [26] is the following: Assume that there are a series of points *x*_0_, *x*_1_, ⋯, *x*_*n*_, and *a* = *x*_0_ < *x*_1_ < ⋯ < *x*_*n*_ = *b*, *y*_*i*_ = *f*(*x*_*i*_) and ${y_{i}}^{\prime }=f^{\prime }\left(x_{i}\right)\;\left(i=0,1,\cdots ,n\right)$ is defined on [*a*, *b*]. If there are functions

$$\begin{array}{c} H\left(x\right)=\left\{ \begin{array}{cc} H_{1}\left(x\right), & x\in [x_{0},x_{1})\\ H_{2}\left(x\right), & x\in [x_{1},x_{2})\\ \cdots \cdots \\ H_{n}\left(x\right), & x\in [x_{n-1},x_{n}) \end{array}\right. \end{array}$$

meet:(1) In each sub range [*x*_*k*_, *x*_*k*+1_](*k* = 0, 1, ⋯, *n*), *H*(*x*) is the three order polynomial, and *H*(*x*_*i*_) = *y*_*i*_, $H^{\prime }\left(x_{i}\right)={y_{i}}^{\prime }\;\left(i=0,1,\cdots ,n\right)$. (2)*H*(*x*), *H*′(*x*) are continuous on [*a*, *b*]. Then *H*(*x*) is the Hermite function of *f*(*x*) on *x*_0_, *x*_1_, ⋯, *x*_*n*_.

**Fig 7:**
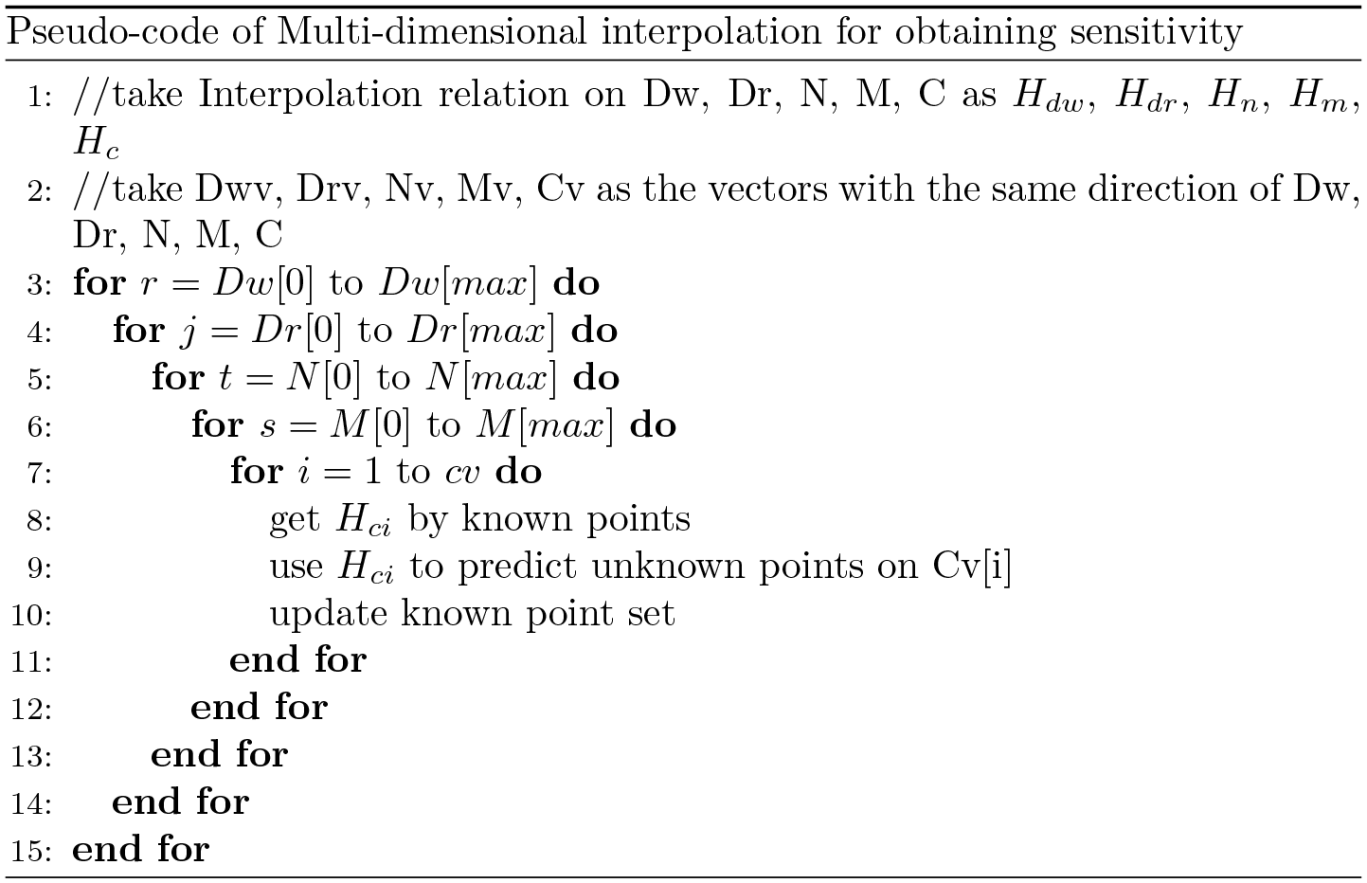
The pseudo-code of multi-dimensional interpolation for obtaining sensitivity. It shows the interpolation in the dimension of CPU. For every vector in the CPU dimension, an interpolation function Hci is trained, and then used to predict unknown points in this vector. After that, the newly predicted points are updated as known points.

The most important work described in this section is to achieve multi-dimensional interpolation by piecewise cubic Hermite interpolation. Multi-dimensional interpolation is the extension of 1-dimensional interpolation and the interpolation should be performed on every effective dimension. In this paper an effective dimension is defined as the dimension in which the resource usage is higher than the lower bound, while a non-effective dimension is defined as the dimension in which the resource usage is below the lower bound. The non-effective dimension means the corresponding resource is used a little or even not used at all by the program. The non-effective dimension can be bypassed during profiling because it will not affect the resource sensitivity value. For the non-effective dimension, we execute the program only once at the lower bound point and use the obtained sensitivity value of that point for all other points of that dimension. Bypassing the non-effective dimension will speed up the profiling process because the time-consuming program execution is avoided. The interpolation in one dimension is conducted in iterative loops in which all other dimensions will go through the predefined resource level points one by one. Interpolation is performed in every effective dimension. The pseudo-code of interpolation in one dimension is shows in Fig 7. It shows the interpolation in the dimension of CPU. For every vector in the CPU dimension, an interpolation function *H*_*ci*_ are trained, and then *H*_*ci*_ are used to predict unknown point in this vector. After that, the newly predicted points are updated as known point. The interpolation in other dimensions uses the similar algorithm as the CPU dimension interpolation.

## Evaluation

### Experiment setup

Table 1 shows the configuration of the server used in our experiment. The server is equipped with two Intel Xeon E5506 (Westmere) processors and 8GB memory, more details can be found in Table 1. The server runs CentOS 6.5 with Linux kernel version 2.6.32.

**Table 1:** Configuration of the server used in experiments.

CPU Type	Intel Xeon E5506
**Cores**	4 cores@2.13G
**Threads per core**	1 thread
**Sockets**	2
**Memory**	8GB, DDR3
**Disk read bandwidth**	1-135 MB/s
**Disk write bandwidth**	1-120 MB/s
**Network bandwidth**	1-100 MBit/s

Table 2 shows the capacity of the five resources and the number of capacity points representing different resource levels. The capacity points are set uniformly within the range of resources provided by the server, for example, 7 points on the number of CPU cores, 24 points on the disk read bandwidth, 21 points on the disk write bandwidth, 13 points on the memory capacity and 17 points on the network bandwidth.

**Table 2:** The capacity and capacity points of the five resources.

Parameters	Control file in Cgroups	Experiment range	Number of capacity point
**Number of cores C**	CPUset.CPUs	2-8	7
**Disk read bandwidth Dr**	blkio.throttle.read_bps_device	20-135 MB/s	24
**Disk write bandwidth Dw**	blkio.throttle.write_bps_device	20-120 MB/s	21
**Memory size M**	memory.limit_in_bytes	2-8GB	13
**Network bandwidth N**	net_cls.classid	20-100MBit/s	17

Several kinds of programs are selected from multiple benchmarks for verifying the effectiveness of our method. Data Caching and Data Serving are selected from Cloudsuite. Different from traditional scale-up parallel programs, Data Caching and Data Serving are scale-out in nature and have complicated resource demand patterns, so they represent the typical workload of data centers [27][28], which is the major target of our study. In order to show the effect of our work in dealing with different workloads, programs with different resource usage patterns should be included in our experiment. For this purpose, more programs are selected from the NAS Parallel Benchmark, PARSEC, and SysBench. All selected programs are listed in Table 3 and categorized into groups according to the resources they demand the most. For different programs, the performance to be optimized and measured can be different, for example, Data Caching emphasizes the average latency, Data Serving concerns the operations per second, and the programs from NAS Parallel Benchmark and PARSEC pursues the instructions per cycle. These different performance criteria are abstracted into performance in the resource sensitivity definitions but different measurement methods are used in the individual profiling experiment.

**Table 3:** Workloads selected.

Suites	Programs	Resource demanded
**Cludsuite**	DataCaching, DataServing	CPU, mem, network bandwidth
**NAS Parallel Benchmark**	mg.C.8, mg.C.4, ft.C.8, ft.C.4	CPU, mem
**PARSEC**	blackscholes, facesim, x264, bodytrack, ferret, freqmine	CPU
**SysBench**	fileiord, fileiowr	disk bandwidth

In order to ensure the accuracy and reality of the evaluation experiments, we repeat an experiment ten times to get average value as the experiment results. Our experiments evaluate the two-level profiling acceleration strategies from different aspects. First, the numbers of iteration step with different profiling acceleration strategies are compared. Second, the accuracies of the prediction models with different sampling methods are evaluated. Third, the effect of *ε* to the sampling set generation is discussed.

### The number of iteration steps

**Fig 8:**
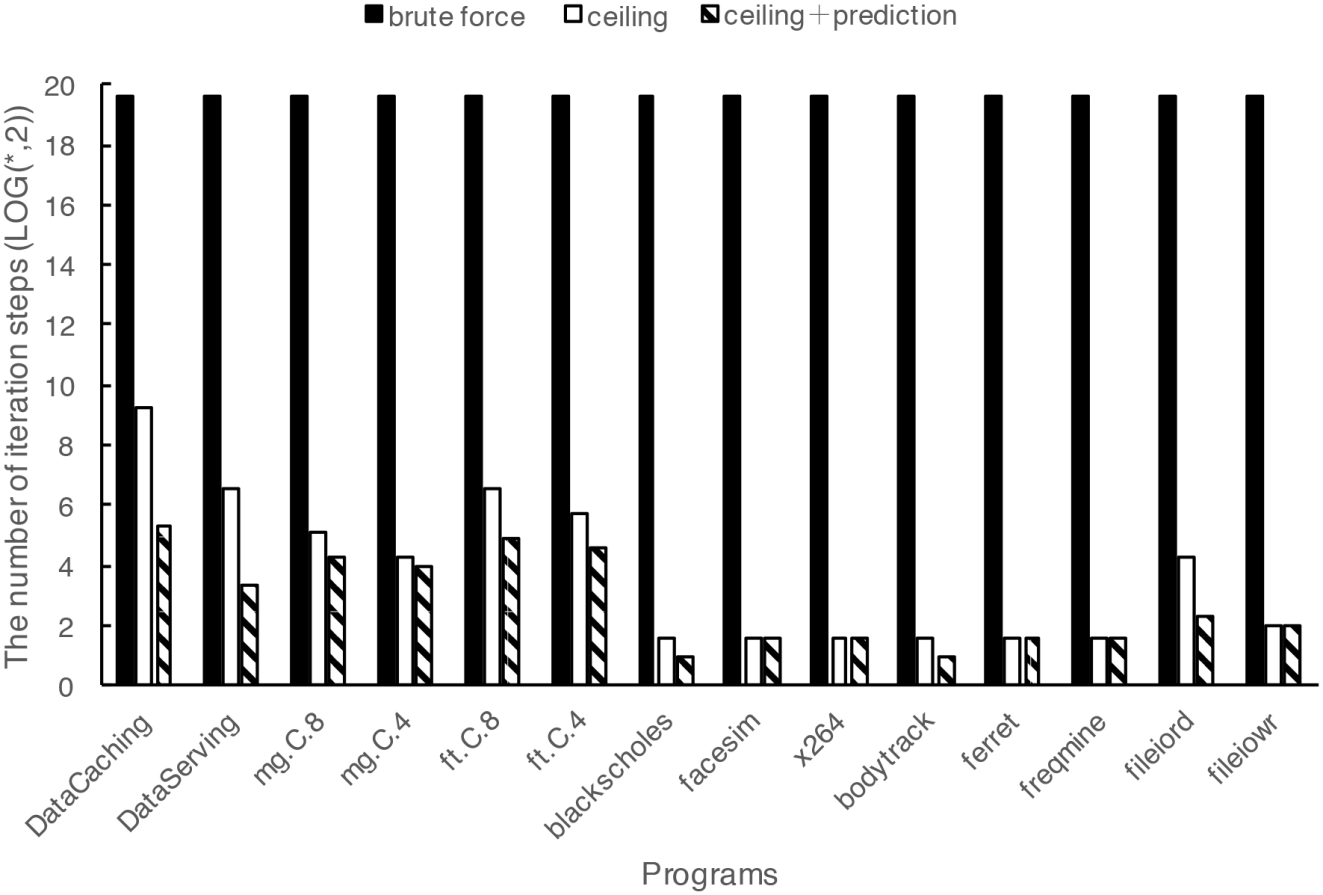
Comparison of the number of iteration steps. The two-level acceleration strategy can significantly reduce the iteration steps and therefore shorten the experiment time. Note, the Y axis is represented in logarithmic scale for clear display.

We use the number of iteration steps in profiling as a criterion to evaluate the efficiency of our fast profiling method. The original brute force strategy for obtaining the resource sensitivity needs to execute the program with all resource combinations. In the case of our resource levels definition, to get the resource sensitivity of a program, the program has to be executed for 7*24*21*13*17 times within the iteration loops of the experiment. As mentioned above, it takes a long time and may not be realistic in many cases. The numbers of iteration steps is reduced by applying the two-level profiling acceleration strategies, which is shown in Fig 8 and Table 4. In Fig 8 the columns represent the number of iteration steps of the original brute force strategy, of the profiling acceleration strategy base on the resource ceiling, and of the profiling acceleration strategy based on both resource ceiling and prediction, respectively. Note, the Y axis values, i.e., the iteration steps, are represented in logarithmic scale for clear display. We can see that both fast profiling strategies, especially the two-level acceleration strategy, significantly reduce the iteration steps and therefore shorten the experiment time. At the same time, we find that programs depending mainly on one resource need fewer iteration steps to complete the experiment than the programs depending on more than one resource. Programs blackscholes, facesim, x264, bodytrack, ferret and freqmine depend mainly on CPU, they need only 2-3 iteration steps to complete the experiment. Program fileiord depends mainly on Disk I/O, it need 5 iteration steps to complete the experiment. Programs mg.C.8, mg.C.4, ft.C.8, ft.C.4 and Data Serving depend mainly on CPU and memory, they need 10-30 iteration steps to complete the experiment. Program Data Caching depends on CPU, memory and network I/O, it needs 40 iteration steps to complete the experiment. We also find that the resource ceiling strategy performs better in reducing the iteration steps for the programs depending mainly on only one resource, while the two-level strategy based on both resource ceiling and prediction model gets better results in the case of programs depending on more than one resource.

**Table 4:** The number of iteration steps.

Programs	Brute force	Ceiling	Ceiling and Prediction
**Data Caching**	779688	595	40
**Data Serving**	779688	91	10
**mg.C.8**	779688	35	20
**mg.C.4**	779688	20	16
**ft.C.8**	779688	91	30
**ft.C.4**	779688	52	24
**blackscholes**	779688	3	2
**facesim**	779688	3	3
**x264**	779688	3	3
**bodytrack**	779688	3	2
**ferret**	779688	3	3
**freqmine**	779688	3	3
**fileiord**	779688	19	5
**fileiowr**	779688	4	4

### The accuracy of interpolation-based acceleration

**Fig 9:**
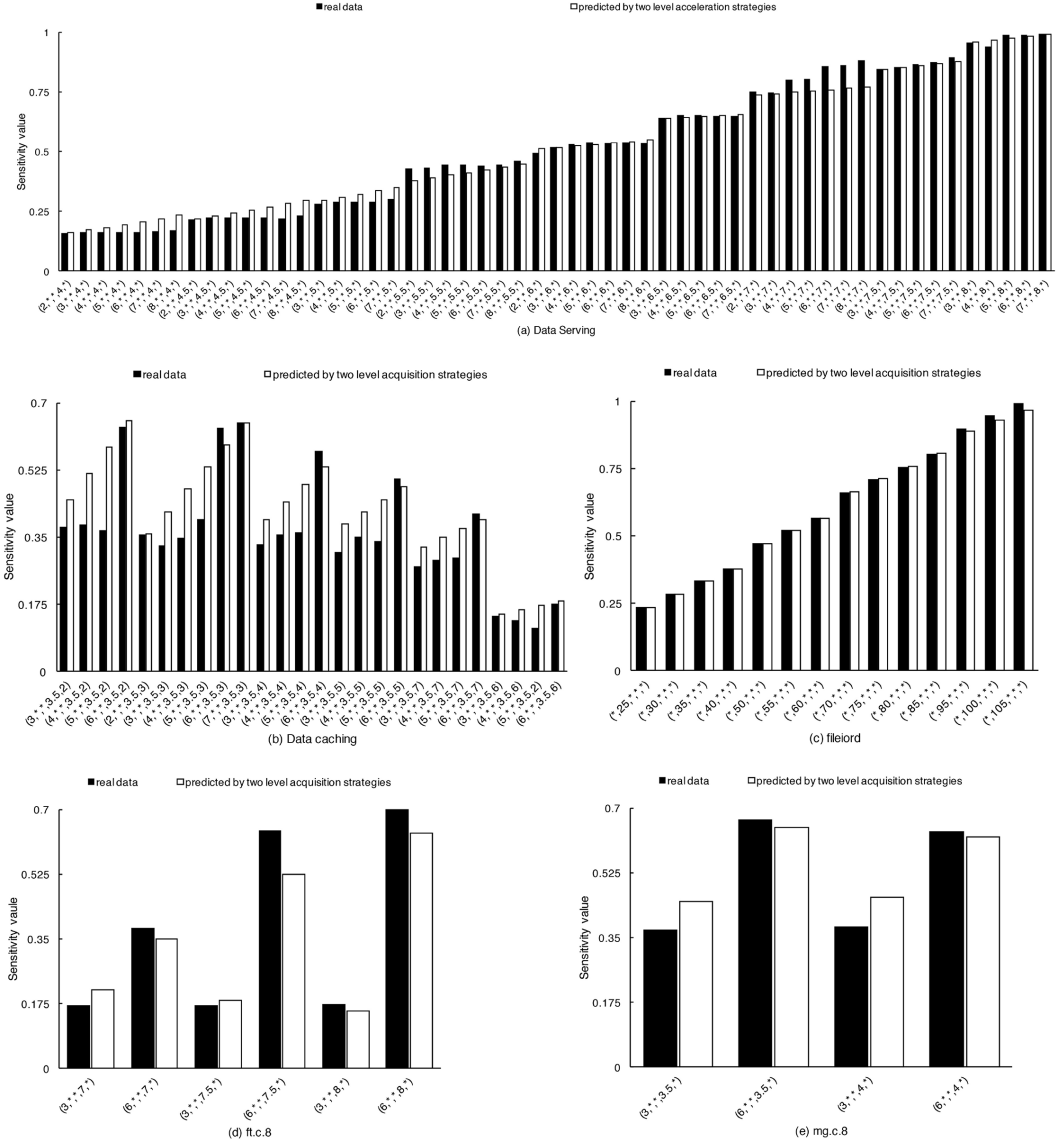
The average absolute error of the interpolation-based acceleration. Fig 9(a), 9(b), 9(c), 9(d) and 9(e) shows the absolute error of the interpolation-based prediction on Data Serving, Data Caching, fileiord, ft.c.8 and mg.c.8. The overall average absolute error of the five programs is 0.038.

In our experiments, the prediction value obtained by our interpolation method is compared with the real value obtained by program execution and the prediction value obtained with other sampling methods. Fig 9 shows the absolute error of the prediction value with the real value. The absolute error is defined as *abs*(*predicted data* − *real data*), and the average absolute error of a program is defined as $\frac{\sum_{i=1}^{N}abs\left(predicted\;data-real\;data\right)}{N}$. The columns represent the real value and the predicted value respectively. The Y axis represents the sensitivity value and the X axis represents the experiment points of different resource conditions represented by Cartesian product. There are five elements in the Cartesian product, they represent CPU, disk read bandwidth, disk write bandwidth, memory and network bandwidth, respectively. Some elements expressed as “*” mean the usage of the corresponding resource is below the low bound of the experiment. Fig 9(a) shows the case of Data Serving. We can see that in most cases the predicted data are smaller than the real data. It is because the curvature of predicted data curve determined by the derivative method of Hermite interpolation is smaller than that of the real data curve, resulting in a relatively flat curve. In the case of Data Serving, the average absolute error of the predicted data from the real data is 0.0257, as shown in Table 5. Fig 9(b) shows the case of Data Caching whose performance is influenced by CPU, memory capacity and network bandwidth. Because there are too many Cartesian product items which are impossible to be shown in the figure, we show only part of the experiment data with memory restriction of 3.5GB, and the average absolute error between the predicted value and the real data is 0.0664. Fig 9(c) shows the case of program fileiord. In this case, the Hermite interpolation curve well fits the real data curve, the average absolute error is very small, only 0.0041. Fig 9(d) and 9(e) show the cases of programs ft.c.8 and mg.c.8 from NPB, respectively. The source of the error in both cases is similar as in the case of DataServer, that is, the curvature of the real data is larger than that of the predicted data. The average absolute errors in the case of ft.c.8 and mg.c.8 are 0.0465 and 0.0472 respectively. The overall average absolute error of the five programs is 0.038.

**Table 5:** The average absolute error of the interpolation-based acceleration.

Programs	The average absolute error
**Data Serving**	0.0257
**Data Caching**	0.0664
**mg.C.8**	0.0472
**ft.C.8**	0.0465
**fileiord**	0.0041
**average**	0.038

**Fig 10:**
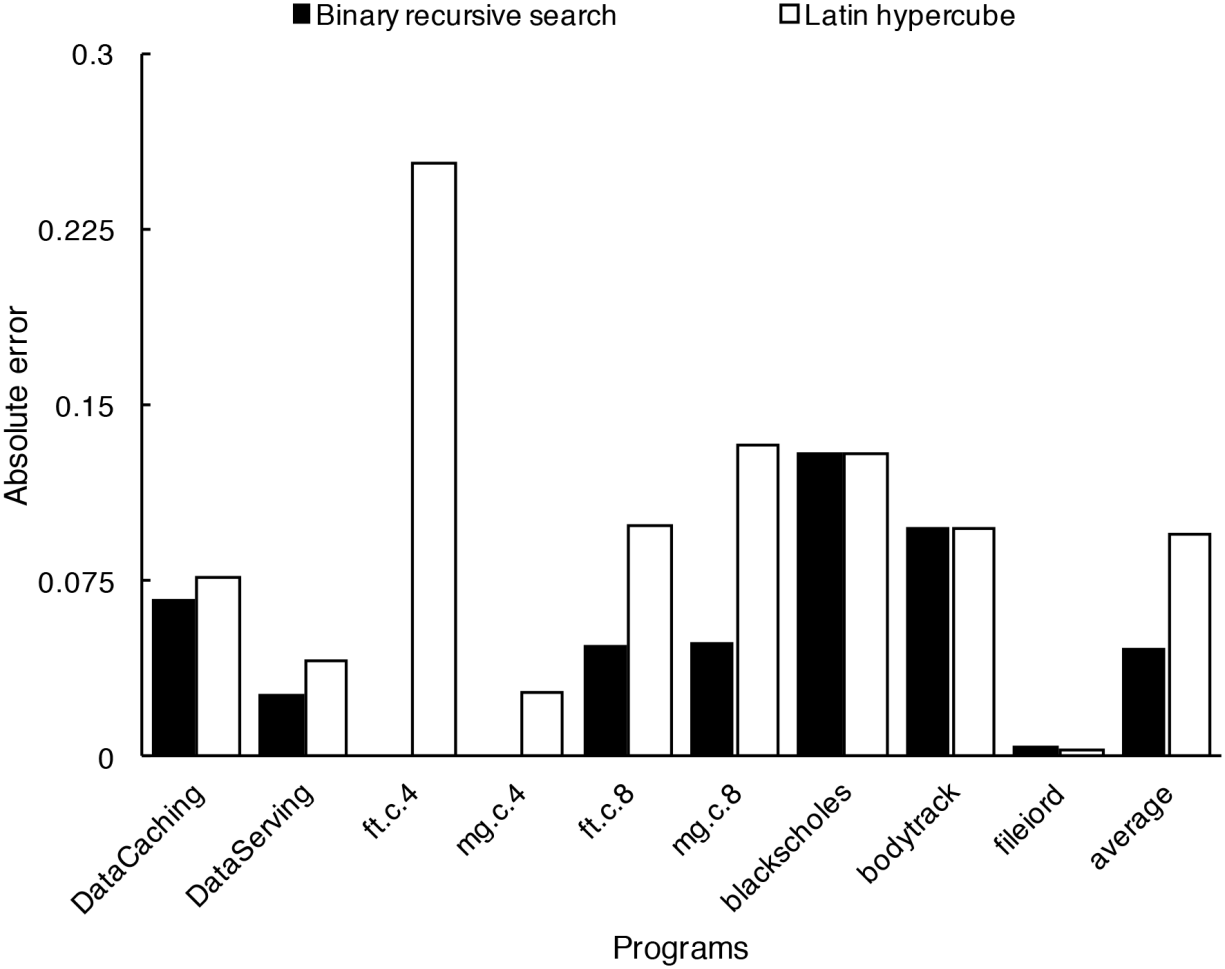
Comparison of methods for generate sampling set. The overall average absolute error of using the sample set by the binary-search-like approach (i.e., 0.0462) is smaller than that of using Latin hypercube sampling (i.e., 0.0953).

The size of the sampling set influences the efficiency of the interpolation-based acceleration strategy. The binary-search-like approach for generating the sampling set is evaluated in our experiments. For comparison, we use Latin hypercube sampling [29] as the reference in generating the sampling set for the five resources. Latin hypercube sampling is a commonly used heuristic method to explore a multi-dimensional parameter space. In order to ensure interpolation, when using Latin hypercube, we put the boundary points into the sampling set and generate other points by Latin hypercube. The number of resource condition points in the two sampling sets generated by the binary-search-like approach and Latin hypercube sampling are kept the same to make the comparison fair. The prediction results based on the two sampling sets are shown in Fig 10. The columns represent the average absolute error with the sampling set generated by the binary-search-like approach and Latin hypercube, respectively. It can be seen that the average absolute error with the sample set generated by the binary-search-like approach in the case of ft.c.4 and mg.c.4 is zero. It is because all nonzero value are in the sample set with the binary-search-like approach. The average absolute error of fileiord by both sample methods is close to zero. It is because the curve of fileiord is almost a straight line, the prediction accuracies of both sampling methods are very good. The overall average absolute error of using the sample set by the binary-search-like approach (i.e., 0.0462) is smaller than that of using Latin hypercube sampling (i.e., 0.0953). The reason is that Latin hypercube sampling is a random sampling, but the binary-search-like approach generates more points in the range with greater performance change and fewer points in the range with small performance change, making points with higher risk of prediction error real value points.

### The effect of *ε*

**Fig 11:**
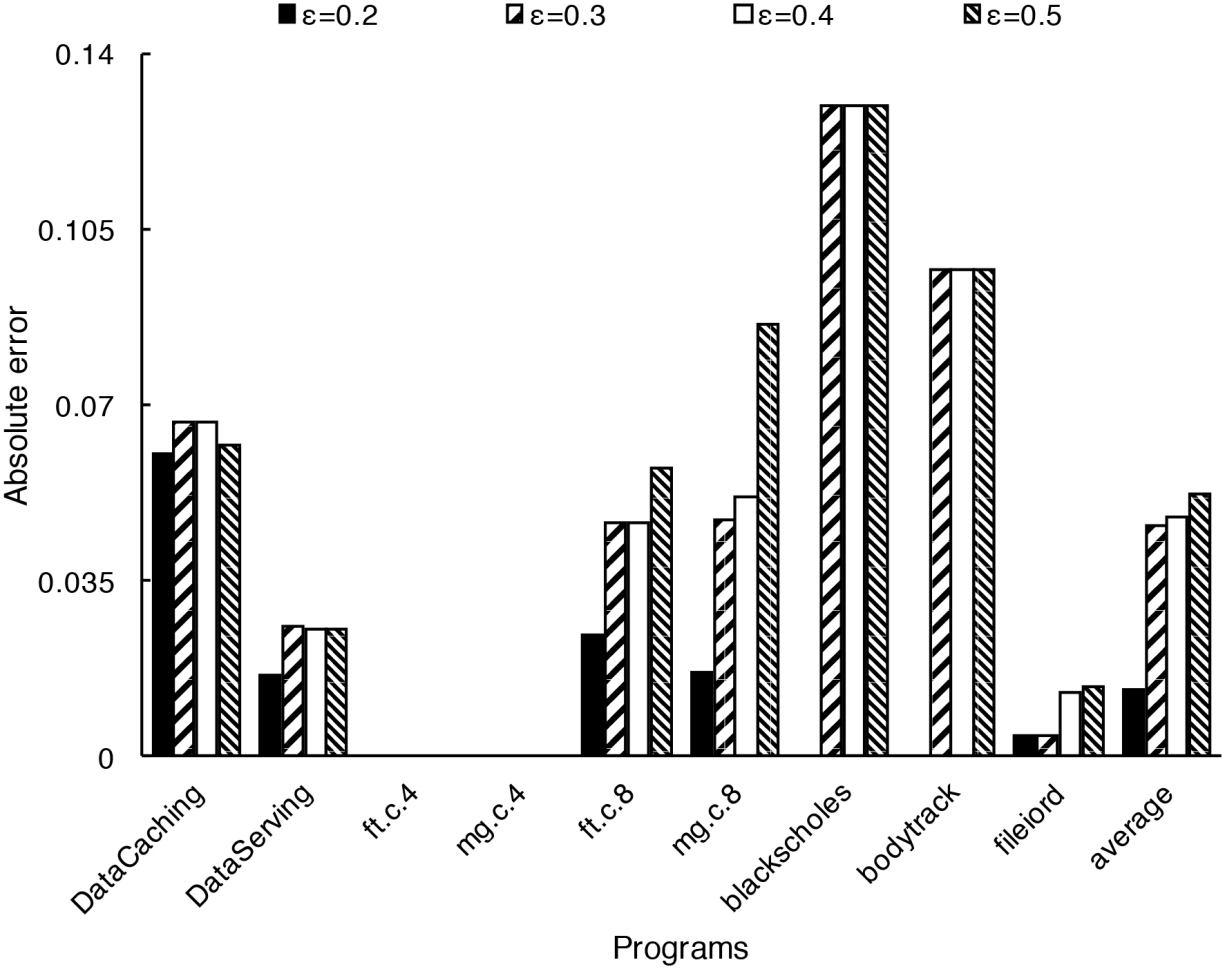
Prediction accuracy with different *ε* value. When *ε* is 0.2, the average absolute error is 0.0456; when *ε* is 0.3, the average absolute error is 0.0851; when *ε* is 0.4, the average absolute error is 0.0866; when *ε* is 0.5, the average absolute error is 0.092.

**Fig 12:**
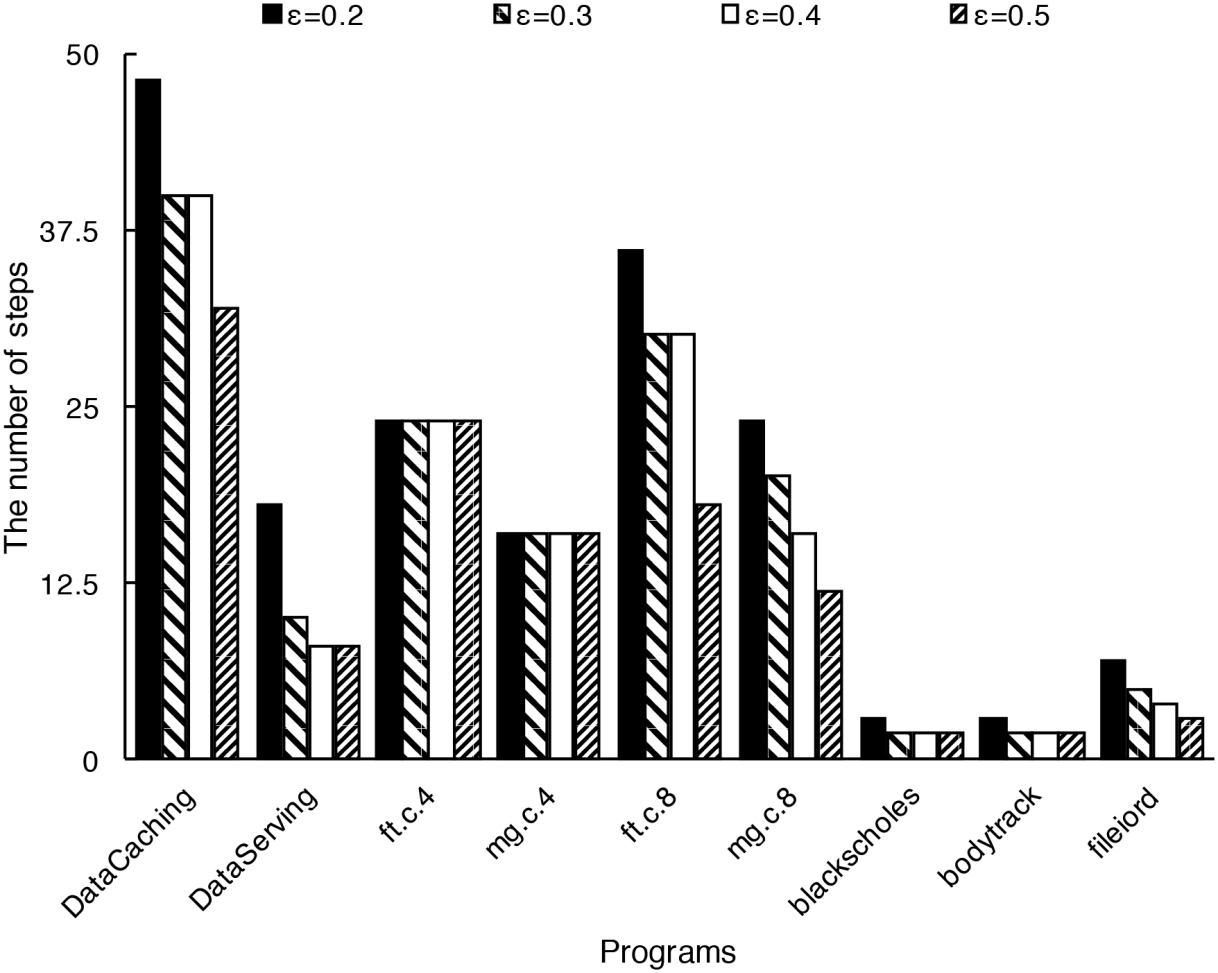
The number of steps for generating the sampling set with different *ε* value. *ε* is chosen as 0.2, 0.3, 0.4, and 0.5, and the number of steps are 179, 149, 142, 117 respectively. The conclusion is that larger *ε* result in fewer number of steps.

*ε* has a direct effect on accuracy. As discussed in 4.1, a larger *ε* will result in a shorter sampling time and a higher absolute error in interpolation. Fig 11 shows the absolute error with different *ε* values, here *ε* is chosen as 0.2, 0.3, 0.4, and 0.5. Fig 12 shows the total number of steps to generate the sampling sets (that is, the number of sampling points) for the benchmark programs with different *ε*. We find when *ε* is 0.2, the accuracy is better, but the number of steps required to generate the sampling set is large. When *ε* is 0.3 and 0.4 the accuracy is moderate. When *ε* is 0.5 the accuracy is low even though the sampling set can be generated quickly. From our experiments, we believe 0.3 or 0.4 might be the proper value of *ε* for most programs.

## Related works

This paper proposes a new metrics system to characterize the program’s performance degradation when it is co-running with other programs, and proposes a two-level profiling acceleration strategy to accelerate the profiling process. This section reviews representative profiling approaches and program characteristics based task scheduling to illustrate the difference between our method and other approaches.

**Methods for profiling program runtime characteristics.** There are a number of profiling tools or methods to obtain program runtime characteristics. The purpose of using the performance profiling tools such as VTune [30], OProfile [31], Gprof [32] is analyzing the hot-spot code and call relationship in the program and locating performance bottleneck. Several OS-level performance profiling tools such as collectl [23], iostat [33] and vmstat [34] are used for analyzing the resource usage of the system. Ferdman et al. [35] introduced the benchmark suite CloudSuite and analyzed the characteristics of those scale-out workloads, finding that today’s predominant processor micro-architecture is inefficient for running those workloads. Jia et al. [36] characterized the micro-architectural characteristics of data analysis applications. They have found that the data analysis applications share many inherent characteristics and presented several recommendations for architecture and system optimizations. Yasin et al. [37] studied the characteristics of a Big Data Analytics workload, aiming at understanding the essential causes of the CPU bottlenecks. Jia [38] studied the workload characteristics with different software stacks and pointed out that the software stacks do have influence to application characteristics. Seo et al. [39] classified application programs based on IO characteristics, and verified the classification with examples. To our best knowledge, there is no mature ruler-like method so far for the cluster or data center operators to measure the performance change of a program when it is co-running with other programs.

**Program characteristics based task scheduling for workload consolidation.** Delimitrou et al. [22, 40] classified application programs respect to scale-up, scale-out, heterogeneity and interference by a technique called collaborative filtering. They map the application programs to proper execution environment to maximize program performance and resource utilization. They have studied how performance varies with different CPU, memory and storage capacity. But the constraint of the collaborative filtering method is that the number of server configurations must be smaller than the number of shot runs, which is much smaller than what we get by our method. Heracle [41] used an isolation mechanism to parallelize the batch tasks and latency-sensitive tasks. But they only explored the program performance variation with a single resource. Mars et al. [42] tested program’s sensitivity to the shared resource on chip instead of shared resource on a server such as disk I/O and network I/O, therefore the method is not relevant to data center workload consolidation.

## Conclusion

Efficient workload consolidation relies on good understanding of the runtime characteristics of co-running programs. With the knowledge of the resource usage and of the relationship between the performance and the resource condition, we could schedule the programs with less resource usage conflict to the same server for execution. The relationship of performance and resources is defined by resource sensitivity. However, getting the resource sensitivity of a program is time consuming, especially when the program relies on multiple resources and each resource is controlled with fine-grain levels. This paper proposes a two-level profiling acceleration strategy for speeding up the process of acquiring the resource sensitivity. The first level of the strategy uses the maximum resource usage, called resource ceiling, as the upper bound of the controlled resource and eliminates the need of performing profiling executions in the whole rage of the resources. The second level of the strategy reduces the number of experiment points further by interpolation and generates the resource sensitivity at the non-sampling points by prediction. Hermite interpolation is used as the prediction model. As far as we know, there has not been a profiling tool for obtaining co-running program characteristics on the resource dimensions of CPU, memory capacity, the disk read/write bandwidth and the network bandwidth. The proposed method is evaluated by comparing the number of steps required by profiling and the difference between the predicted data with the real data, using different acceleration strategies and different sampling methods. The evaluation results show that the Hermite interpolation reduces the number of steps in generating the sampling set. Our method significantly shortens the time for profiling the resource sensitivity of a program while still maintaining a reasonable accuracy.

Our future work will focus on three aspects. The first is to apply our fast profiling method to more versatile programs to see their feasibility in supporting general program profiling. The second is to integrate the resource sensitivity information into the scheduler for workload consolidation, and use the scheduler to schedule real workloads to verify its effectiveness in improving system throughput, resource utilization and program performance. The third is to expand our method to cover more server configurations. Since in a modern data center there are multiple types of servers and different server configurations, adapting our method to different server types and configurations is necessary for its real usage in the data center.
